# Effect of Filtration and Thickness of Cross-Sections of Cone Beam Computed Tomography Images on Detection of Proximal Caries

**Published:** 2017-07

**Authors:** Mehrdad Abdinian, Rahman Nazeri, Marzieh Ghaiour

**Affiliations:** 1 Assistant Professor, Dental Implants Research Center, Department of Oral and Maxillofacial Radiology, School of Dentistry, Isfahan University of Medical Sciences, Isfahan, Iran; 2 Postgraduate Student, Department of Research, School of Dentistry, Isfahan University of Medical Sciences, Isfahan, Iran; 3 Postgraduate Student, Department of Pediatric Dentistry, School of Dentistry, Isfahan University of Medical Sciences, Isfahan, Iran

**Keywords:** Dental Caries, Radiography, Tomography, Diagnosis

## Abstract

**Objectives:**

When a patient has cone beam computed tomography (CBCT) images based on the treatment plan, it is possible to use these images for evaluation of caries, and there is no need for new radiographs, according to the “as low as reasonably achievable” (ALARA) principle. The aim of this study was to determine the effect of filtration and thickness of CBCT cross-sections on detection of proximal caries.

**Materials and Methods:**

In this in-vitro study, 100 teeth were placed in the dental sockets of a dry skull, and were fixed in normal proximal contacts. CBCT images were taken and were evaluated by two observers on the panoramic view at 1-, 3- and 5-mm-thick cross-sections, with the use of filtrations 0, 1 and 2. Afterwards, the samples were sectioned and underwent a histological evaluation. McNemar’s test was used to compare the findings on CBCT images and histological evaluation. Receiver operating characteristic (ROC) curves and logistic regression were used to evaluate the diagnostic accuracy of different cross-sections.

**Results:**

The maximum AZ-value was achieved at 3-mm thickness/filtration 2. However, the differences between 1-mm thickness/filtration 2 and 1-mm thickness/filtration 1 were not significant (P=0.728 and 0.868, respectively). The minimum AZ-value was achieved at 5-mm thickness/filtration 0.

**Conclusions:**

Although CBCT is not sufficiently effective in detecting caries, the best cross-sections for detection of proximal caries were achieved at 3-mm thickness/filtration 2, 1-mm thickness/filtration 2 and 1-mm thickness/filtration 1.

## INTRODUCTION

Dental caries is the most prevalent clinical problem in dentistry and has a high incidence rate in the population [1]. Therefore, dentists should be able to make a correct and early diagnosis of dental caries. It is necessary to have a proper knowledge of the depth of caries in order to prepare an appropriate treatment plan [2,3]. Clinical and visual examinations, illumination and intraoral radiography with conventional and digital radiographic techniques are the most commonly used diagnostic methods implemented by dentists to detect caries [4–6].

However, diagnosis of interproximal caries in posterior teeth is still a challenge in dentistry, and it usually requires a combination of clinical and radiographic examinations [1,7]. In recent years, conventional bite-wing radiography has been the most commonly used technique to this end [8,9]. Digital radiography systems were introduced in early 1990. These systems make it possible to manipulate image contrast and brightness to improve the diagnostic quality of the image [10]. One of these digital systems is cone-beam computed tomography (CBCT).

This new technique helps dental practitioners to collect data and improve treatment planning with the use of three-dimensional (3D) images at axial, sagittal and coronal cross-sections [11]. CBCT technique is used in different fields of dentistry [4]. Although the use of CBCT images for detection of proximal caries has previously been evaluated, there are still doubts about the superiority of CBCT systems over conventional systems [5, 12–19]. However, based on a search by the authors of the present study, no studies to date have evaluated the effect of the thickness of cross-sections and filtration on the accuracy of caries detection on CBCT images. Therefore, the present study was undertaken to evaluate the diagnostic accuracy of CBCT systems for detection of proximal caries, and also to evaluate the effect of cross-section thickness and filtration (resolution) of images on detection of proximal caries, with the null hypothesis that CBCT images are not accurate enough for detection of caries.

## MATERIALS AND METHODS

**Fig. 1:**
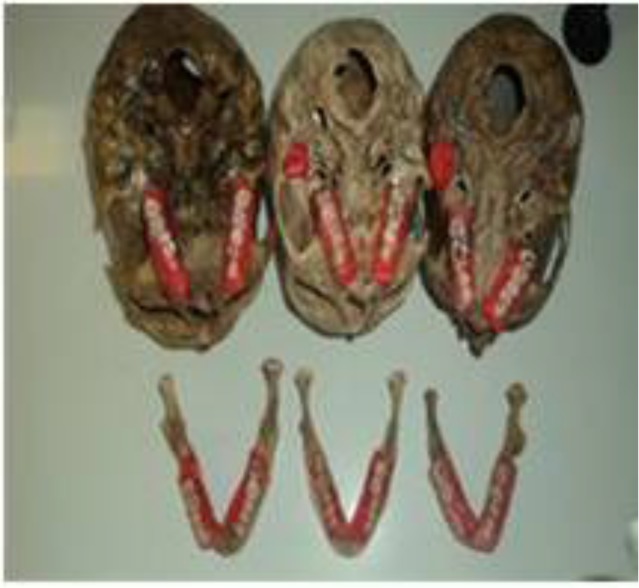
Placement of the teeth in a normal anatomical position

In the present in-vitro study, 100 human teeth, consisting of 20 canines, 40 premolars and 40 molars with or without proximal caries were selected from among 400 teeth, using simple random technique. The teeth had been extracted for periodontal or orthodontic reasons at the School of Dentistry, Isfahan University of Medical Sciences. The teeth were cleaned of all the calculi and debris using an ultrasonic device (Cavitron JEJ Plus Ultrasonic Scaler, Dentsply, Milford, USA), were disinfected in 2% sodium hypochlorite (NaOCl) solution for 20 minutes, and were stored in distilled water. Then, the crowns were separated from the roots using a #835 bur (0.8, Tizkavan, Iran). The dental crowns were divided into 20 groups (n=5). Ten groups included maxillary teeth, and the rest included mandibular teeth. Each group consisted of a canine, first and second premolars and first and second molars in one quadrant. Five dry skulls were prepared, and 4 groups of teeth were placed in the alveolar sockets of each skull so that the teeth in each group were placed in their real anatomical positions, with proper proximal contacts in the maxillary and mandibular alveolar sockets. Finally, the teeth were fixed in their sockets with red utility wax. The placement was done in a way that each row was in a good proximal contact. The mandibular and maxillary teeth were occluded together, and were fixed by wax (this positioning simulates a normal anatomical position), (Fig. 1). To simulate soft tissues and the effect of their distribution, an acrylic resin block, measuring 14.5 mm in thickness, was placed in front of the teeth [20]. Subsequently, the mesial and distal surfaces of the five teeth in each group were evaluated for caries; a total of 200 surfaces in 100 teeth were evaluated.

**Fig. 2:**
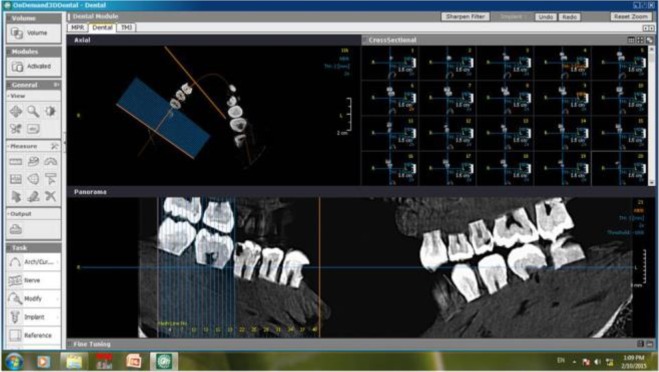
CBCT image of the skull holding the teeth

In the next stage, CBCT images were taken with the use of Soredex CBCT unit (Helsinki, Finland). Each skull was fixed in the CBCT system with the use of ear rods and was scanned for 12.6 seconds at kilovoltage peak (kVp)=89 and milliampere (mA)=6. The images were reconstructed with the system’s software program (OnDemand 3D Dental 1.0.9.1343). Figure 2 shows a CBCT image of the skull holding the teeth.

The images were evaluated by two oral and maxillofacial radiologists with 6 years of experience in a random single-blind manner. The images were visualized on a 22-inch 32-bit LG monitor (LG, Seoul, Korea) with a resolution of 1440×690 pixels, using the CBCT system’s software program in a dimly lit room. The observers scored the images on the panoramic view (mesiodistal) with three different filtrations (resolutions) of 0, 1 and 2, and three different cross-section thicknesses of 1, 3 and 5 mm, based on the presence or absence of proximal cares in a 5-point scale [21], as follows:

1. Definitively carious
2. Possibly carious
3. Not definitive and suspected
4. Possibly not carious
5. Definitively not carious

In order to evaluate intra-observer agreement, the observers evaluated and scored the images twice with an interval of two weeks, in order to eliminate the effect of memory. Subsequently, the teeth were sectioned mesiodistally, parallel to the tooth long axis with a mean cross-section thickness of 0.4mm, using Accutom cutting instrument (Struers, Ballerup, Denmark).

Each cross-section was evaluated by a pathologist under a stereomicroscope at ×15 magnification. Each white demineralized lesion or yellow-brown discoloration in enamel or dentin was considered a carious lesion in the histopathological view [5]. Of all the cross-sections of each tooth, one cross-section with the greatest depth was selected for scoring of that tooth based on the following scoring system:

1. No caries in the proximal surface
2. Proximal caries in enamel
3. Proximal caries extending to the dentino-enamel junction (DEJ) or located in the external half of dentin

Kappa agreement coefficient was calculated for intra- and inter-observer agreement, and was classified as follows:

0.10 <: No agreement
0.11–0.4: Poor agreement
0.41–0.60: Acceptable agreement
0.61–0.80: Strong agreement
0.81–1.0: Excellent agreement

In order to evaluate the accuracy of caries detection in different radiologic cross-sections, the results reported by the radiologists were compared with the histopathology findings using complete and absolute sensitivity, specificity and the receiver operating characteristics (ROC) analysis. Values under the ROC curve (AZ-values) were calculated for different cross-sections using SPSS 22 software program (IBM Co., Chicago, IL, USA). Wilson confidence interval-based comparison was used to evaluate the differences in the accuracy of different cross-sections (α = 0.05).

## RESULTS

Table 1 presents the histopathologic status of 200 proximal dental surfaces. According to this Table, 27% of the surfaces were not carious, while carious lesions were detected in 73% of the surfaces. At different cross-section thicknesses and filtrations, intra-observer kappa coefficients of observers 1 and 2 were in the ranges of 0.759–0.884 and 0.716–0.867, respectively. However, inter-observer kappa coefficient was in the range of 0.631–0.769, which was calculated based on the means of data provided by each observer for each parameter. A high intra-observer kappa coefficient indicates a strong intra-observer agreement. Therefore, our calculations were made by considering the first readings of each observer. Table 2 shows the results of sensitivity and specificity of different thicknesses and filtrations.

**Table 1.** Percentage of carious lesions in each dental region

**Caries Situation**	**Frequency**	**Percentage**
Without caries	54	27
Caries in enamel	57	28.5
Caries in the external half of dentin	48	24
Caries in the internal half of dentin	41	20.5

**Table 2.** Absolute and complete sensitivity and specificity of caries detection at different thicknesses and filtrations

**Thickness (mm)**	**Filtration**	**Absolute sensitivity**	**Complete sensitivity**	**Absolute specificity**	**Complete specificity**
1	0	0.556	0.667	0.644	0.719
	1	0.130	0.815	0.260	0.493
	2	0.556	0.686	0.555	0.692
2	0	0.074	0.500	0.219	0.431
	1	0.500	0.778	0.479	0.616
	2	0.648	0.759	0.616	0.719
3	0	0	0.167	0.144	0.267
	1	0.093	0.389	0.295	0.507
	2	0.278	0.537	0.493	0.637

**Fig. 3:**
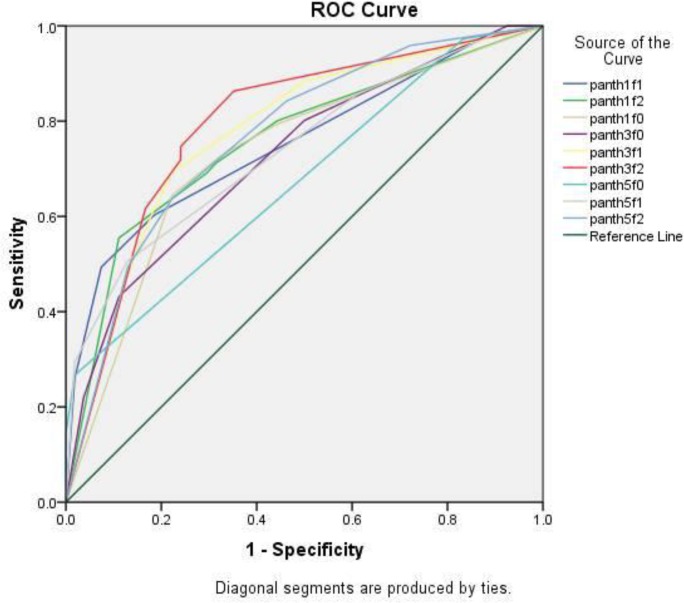
ROC curve of different filtrations and thicknesses (pan th1f0= thickness 1 filtration 0, pan th1f1=thickness 1 filtration 1, pan th1f2= thickness 1 filtration 2, pan th3f0= thickness 3 filtration 0, pan th3f1=thickness 3 filtration 1, pan th3f2= thickness 3 filtration 2, pan th5f0= thickness 5 filtration 0, pan th5f1=thickness 5 filtration 1, pan th5f2= thickness 5 filtration 2)

The results indicated a direct correlation between filtration and correct diagnosis in CBCT, and a reverse correlation between the thickness of view and correct diagnosis in CBCT. Figure 3 presents the ROC curve of different filtration and thicknesses according to the histopathological results. Table 3 shows the AZ-value of different views resulted from the ROC curve. The results showed a direct correlation between the histopathological and radiologic results. Based on the P-values presented in Table 3, AZ-values of all the cross-sections and filtrations were significantly higher than the 0.5 reference point of the AZ-value (P<0.05). The highest AZ-value was achieved at 3-mm thickness/filtration 2, with the lowest AZ-value at 5-mm thickness/filtration 0. Although the AZ-values of different filtrations and thicknesses were almost the same, based on Wilson test, the best view was achieved at 3-mm thickness/filtration 2, which exhibited significant differences in diagnostic accuracy from

1-mm thickness/filtration
0 (P<0.001), 3-mm thickness/filtration
0 (P<0.001), 3-mm thickness/filtration
1 (P<0.001), 5-mm thickness/filtration
1 (P<0.001) and 5-mm thickness/filtration
2 (P=0.046).

**Table 3.** Az-values, standard errors (SE) and significance levels of data

**Test Value Cut-off**	**Az-value**	**SE**	**P-value (asymptomatic sig.)**
TH1/F0	0.731	0.040	<0.001

TH1/F1	0.752	0.035	<0.001
TH1/F2	0.758	0.037	<0.001
TH3/F0	0.726	0.039	<0.001
TH3/F1	0.775	0.038	<0.001
TH3/F2	0.794	0.038	<0.001
TH5/F0	0.673	0.040	.007
TH5/F1	0.740	0.036	<0.001
TH5/F2	0.768	0.038	<0.001

TH =Thickness, F=Filtration

However, there were no significant differences between 3-mm thickness/filtration 2 and 1-mm thickness/filtration 1 (P=0.617), or between 3-mm thickness/filtration 2 and 1-mm thickness/filtration 2 (P=0.459). Table 4 shows the means of correct diagnoses of carious lesions at different thicknesses and filtrations. According to this Table, the most accurate diagnoses were related to the teeth with carious lesions in the external half of dentin, followed by carious lesions in the internal half of dentin.

**Table 4.** Average percentage of the correct diagnoses (accuracy) of the two observers at different views

**Test Value Cut-off**	**Without caries**	**Caries in enamel**	**Caries in the external half of dentin**	**Caries in the internal half of dentin**
TH1/F0	81.4	12.2	62.5	85.3
TH1/F1	68.5	42.1	81.2	92.6
TH1/F2	66.6	45.6	85.4	92.6
TH3/F0	50	12.2	50	78.0
TH3/F1	77.7	31.5	81.2	87.8
TH3/F2	75.9	45.6	87.5	90.2
TH5/F0	16.6	7	22.9	58.5
TH5/F1	38.8	29.3	56.2	78
TH5/F2	53.7	38.5	75	85.3

TH =Thickness, F=Filtration

The most inaccurate diagnoses in all the cross-sections were related to the teeth with enamel caries except for 1-mm thickness/filtration 0, in which caries-free teeth were more accurately diagnosed compared to the teeth with carious lesions in the external half of dentin.

## DISCUSSION

CBCT is a new imaging technique in dentistry. All the new diagnostic methods should be compared with the imaging techniques that are currently used in the clinic. Although there are some studies available in this regard, there are still some debates in relation to the efficacy of the CBCT technique [14].

Akdeniz et al [12] reported that CBCT might prove promising for monitoring small carious lesions. Tsuchida et al [13] reported that the 3D Accuitomo CBCT system was not accurate enough for detection of proximal caries in comparison to conventional film radiography.

An in-vitro study by Haiter-Neto et al [14] showed a reverse correlation between the size of the field of view (FOV) and diagnostic efficacy. Qu et al [22] stated that the type of CBCT system and the size of the FOV have no effect on the diagnostic accuracy of proximal caries.

Charuakkra et al [11] reported that axial cross-sections are the most favorable views to study proximal caries. Based on a search carried out by the authors of the present study, no studies to date have evaluated the effect of cross-section thickness and filtration on the efficacy of CBCT images in detecting proximal caries. In the present study, both inter- and intra-observer coefficients were high (strong to excellent), indicating that the observers had been trained well and were calibrated. The results of the logistic regression of different views showed a direct correlation between filtration and correct diagnosis in CBCT, and a reverse relationship between the thickness of views and correct diagnosis in CBCT.

Therefore, the increase of thickness decreases the number of correct diagnoses, whereas the increase of filtration, increases the number of true diagnoses in CBCT. In this study, ROC curves were used to evaluate the diagnostic accuracy of different cross-section thicknesses and filtration rates. ROC analysis is a more comprehensive parameter for evaluation of diagnostic accuracy compared to sensitivity and specificity [23].

In addition, ROC analysis is a more eloquent parameter for comparison of the diagnostic performance of two or more diagnostic techniques, because it is less significantly affected by the inherent ability of the observer to interpret images, and has been used in various studies [23,24]. In the present study, the AZ-value range was 0.67–0.79. To interpret this finding, it should be pointed out that values approaching 1 indicate a proper test, and values approaching 0.5 indicate poor results of the test [25]. According to the AZ-values in the present study, CBCT technique cannot be considered an accurate tool for evaluation of caries. AZ-value of 3-mm thickness/filtration 2 was significantly higher than that of the other thicknesses; however, there were no significant differences between the cross-sections mentioned above and the two cross-sections with 1-mm thickness/filtration 1 and 1-mm thickness/filtration 2. Therefore, these views were the best views for evaluation of proximal caries. In a study by Zhang et al [15], AZ-values were calculated near the chance value (0.5) with the use of Promax 3D and Kodak 3D900 systems [15]. In studies carried out by Zhang et al [15] and Haiter-Neto et al [14], the AZ-value for diagnosis of incipient proximal caries was in the 0.59–0.64 range. The low AZ-value in these studies might be explained by the fact that evaluations were carried out on incipient enamel carious lesions. However, AZ-values in some other studies [13,21] were higher than that in the present study. In studies by Belem et al [21], and Kayipmaz et al [17], the AZ-values were 0.87 and 0.84, respectively. Another study with high AZ-values has been performed by Charuakkra et al [11]. High AZ-values in these studies might be attributed to the thin CBCT cross-sections of dental structures, yielding a high contrast between carious lesions and sound dental structures that resulted in fewer misinterpretations; as a result, incorrect diagnoses decreased, while AZ-values increased. In the present study, low AZ-values might be attributed to the thickness of cross-sections; therefore, some carious lesions were left undetected. Based on the results of the present research, the highest AZ-value was related to 3-mm thickness, which might be explained by the fact that there is an increase in the noise of cross-sections as the cross-section thickness decreases; on the other hand, with an increase in thickness, diagnosis of caries becomes more difficult due to the effect of partial volume averaging. Therefore, medium thicknesses show proximal caries better than other thicknesses. It is necessary to simultaneously evaluate sensitivity and specificity to compare the diagnostic accuracy of different diagnostic views [25]. Analysis of the ROC curve and AZ-values properly achieve this aim [26, 27]. In the present study, the AZ-values were almost similar in different cross-sections; however, the highest values were related to 3-mm thickness/filtration 2, 1-mm thickness/filtration 1 and 1-mm thickness/filtration 2. Therefore, these views can be considered the best views for diagnosis of caries among all the views evaluated in the current study. Considering the “as low as reasonably achievable” (ALARA) principle, there should be a strong justification for the use of CBCT system before performing any other radiographic examination, and given the low AZ-values of this system, the evidence-based selection criteria should be considered. In this context, the present study did not support the use of CBCT system for diagnosis of caries. One of the limitations of the present study was the fact that only the panoramic view of the CBCT system was evaluated, while axial views were not assessed.

In addition, a limited number of cross-sections was evaluated in the present study to detect caries. Another limitation of the present study was the fact that this study has been carried out in vitro under ideal conditions, and non-moving objects were radiographed in the absence of metallic restorations and tissues around the teeth, which might complicate detection of caries in vivo.

## CONCLUSION

Within the limitations of the present study, it has been concluded that CBCT technique is not accurate enough for evaluation of proximal caries. The best cross-sections for evaluation of caries were achieved at 3-mm thickness/filtration 2, 1-mm thickness/filtration 1 and 1-mm thickness/filtration 2.
